# A Modified Public Health Automated Case Event Reporting Platform for Enhancing Electronic Laboratory Reports With Clinical Data: Design and Implementation Study

**DOI:** 10.2196/26388

**Published:** 2021-08-11

**Authors:** Ninad Mishra, Jon Duke, Saugat Karki, Myung Choi, Michael Riley, Andrey V Ilatovskiy, Marla Gorges, Leslie Lenert

**Affiliations:** 1 Division of STD Prevention National Center for HIV/AIDS, Viral Hepatitis, STD, and TB Prevention Centers for Disease Control and Prevention Atlanta, GA United States; 2 Center for Health Analytics and Informatics Georgia Tech Research Institute Atlanta, GA United States; 3 Biomedical Informatics Center Medical University of South Carolina Charleston, SC United States

**Keywords:** public health surveillance, sexually transmitted diseases, gonorrhea, chlamydia, electronic case reporting, electronic laboratory reporting, health information interoperability, fast healthcare interoperability resources, electronic health records, EHR

## Abstract

**Background:**

Public health reporting is the cornerstone of public health practices that inform prevention and control strategies. There is a need to leverage advances made in the past to implement an architecture that facilitates the timely and complete public health reporting of relevant case-related information that has previously not easily been available to the public health community. Electronic laboratory reporting (ELR) is a reliable method for reporting cases to public health authorities but contains very limited data. In an earlier pilot study, we designed the Public Health Automated Case Event Reporting (PACER) platform, which leverages existing ELR infrastructure as the trigger for creating an electronic case report. PACER is a FHIR (Fast Health Interoperability Resources)-based system that queries the electronic health record from where the laboratory test was requested to extract expanded additional information about a case.

**Objective:**

This study aims to analyze the pilot implementation of a modified PACER system for electronic case reporting and describe how this FHIR-based, open-source, and interoperable system allows health systems to conduct public health reporting while maintaining the appropriate governance of the clinical data.

**Methods:**

ELR to a simulated public health department was used as the trigger for a FHIR-based query. Predetermined queries were translated into Clinical Quality Language logics. Within the PACER environment, these Clinical Quality Language logical statements were managed and evaluated against the providers’ FHIR servers. These predetermined logics were filtered, and only data relevant to that episode of the condition were extracted and sent to simulated public health agencies as an electronic case report. Design and testing were conducted at the Georgia Tech Research Institute, and the pilot was deployed at the Medical University of South Carolina. We evaluated this architecture by examining the completeness of additional information in the electronic case report, such as patient demographics, medications, symptoms, and diagnoses. This additional information is crucial for understanding disease epidemiology, but existing electronic case reporting and ELR architectures do not report them. Therefore, we used the completeness of these data fields as the metrics for enriching electronic case reports.

**Results:**

During the 8-week study period, we identified 117 positive test results for chlamydia. PACER successfully created an electronic case report for all 117 patients. PACER extracted demographics, medications, symptoms, and diagnoses from 99.1% (116/117), 72.6% (85/117), 70.9% (83/117), and 65% (76/117) of the cases, respectively.

**Conclusions:**

PACER deployed in conjunction with electronic laboratory reports can enhance public health case reporting with additional relevant data. The architecture is modular in design, thereby allowing it to be used for any reportable condition, including evolving outbreaks. PACER allows for the creation of an enhanced and more complete case report that contains relevant case information that helps us to better understand the epidemiology of a disease.

## Introduction

### Background

Public health surveillance—the ongoing and systematic collection, analysis, and interpretation of health-related data essential to planning, implementation, and evaluation of public health practice [1]—has been the cornerstone of public health practice. These data are important for understanding the burden, trends, pattern, and general epidemiology of diseases. These requirements for reporting diseases are mandated by state laws or regulations. In 1990, the Council for State and Territorial Epidemiologists (CSTE) and the US Centers for Disease Control and Prevention collaborated to develop uniform criteria to author case definitions and to facilitate reporting of nationally notifiable diseases [2]. To qualify a case as reportable, it must meet the case definition for the condition [3]. These case definition criteria are based on laboratory information, clinical observations, or both. Previously, notifiable diseases were manually reported to public health agencies. Advancements in data and messaging standards have led to the modernization of this process by electronically reporting cases from either electronic health records (EHRs) via electronic case reporting (eCR) or laboratories via electronic laboratory reporting (ELR) [4]. Health level 7 (HL7)–based messages for ELR and eCR have been developed as tools to facilitate case reporting, alleviate the burden of reporting from clinical providers, and establish communication between often disparate EHR systems [5]. Electronic laboratory reports are created on the HL7 version 2 specification [6]. ELR has been increasing in adoption and is a reliable source of reporting cases to public health authorities [7] but contains little information about a case other than the test result. This information, such as demographics, diagnoses, and treatment, is important to public health to understand disease epidemiology and typically exists within the EHR. Similarly, existing eCR architectures are created on the HL7 Consolidated Clinical Document Architecture (C-CDA) [8]. Although eCR is triggered from the EHR, treatment information is not always available at the time the electronic case report is created, which typically occurs earlier on in the continuum of care when the triggering criteria have been met for an automated case report to be sent to public health authorities. Demographic information is important to understand the epidemiology, which, in turn, informs the policies and strategies for the prevention and control of diseases. Treatment information is particularly important at this point in time because of the worrisome trend of antimicrobial resistance exhibited in gonorrhea cases. To address this emerging threat of antimicrobial-resistant gonorrhea and to ensure that patients receive the highest quality of care, monitoring of treatment practices is a critical public health priority [9].

Improvements in interoperability and automation are crucial for public health surveillance [10]. Health care data standards have been developed to reduce ambiguity and facilitate interoperability among health care providers, public health, and other stakeholders [11]. Advances have been made by messaging standards developed by global leaders, such as the HL7. Furthermore, the adoption of FHIR (Fast Health Interoperability Resources), an HL7 standard, has moved semantic interoperability forward by leaps over the last several years [12]. FHIR are easy to implement and leverage the latest web data exchange standards while using existing data standards such as Logical Observation Identifiers Names and Codes (LOINC) [13]; Systematized Nomenclature of Medicine—Clinical Terms [14]; and International Classification of Diseases, Tenth Revision, Clinical Modification [15]. FHIR *Resources* are building blocks that contain a common way to define and represent concepts, sometimes from external terminologies or ontologies (Systematized Nomenclature of Medicine—Clinical Terms, RxNorm, LOINC, etc), a common set of metadata, and a human-readable component [16]. In addition, Clinical Quality Language (CQL), an HL7 authoring standard, has been developed to harmonize standards used for authoring clinical decision support and clinical quality measurement artifacts [17]. CQL is intended to be human-readable and enables queries into EHRs via a set of curated logics. In an earlier pilot, we successfully demonstrated a FHIR-based case reporting at the Indiana Health Information Exchange, which automatically created electronic case reports in 84.6% of the cases [18]. This architecture of eCR extracted all clinical encounters associated with the patient, resulting in a large amount of redundant data that were not associated with that particular episode of infection. It was evident that we had to not only filter information related to the particular condition but also to allow health systems to have control over what type of data are reported to public health authorities.

### Objectives

In this study, we evaluated the pilot implementation of a modified FHIR-based approach for eCR that leverages electronic laboratory reports as triggers to capture relevant expanded case data from EHRs. We describe how this interoperable approach, which is based on open-source and widely adopted health care data standards, can bridge public health and health care data systems by providing relevant, timely, and substantially more information on reportable cases. This implemented architecture, which is known as Public Health Automated Case Event Reporting (PACER), leverages not only FHIR protocols but also the HL7 CQL for interoperable query design. This modified architecture is specifically designed to ensure that health systems have complete control over their data that are used for public health reporting by configuring a set of FHIR resources and data elements that can be removed with this *FHIR Filter*. The modular and versatile components that form the architecture connect to the EHR and extract data as an electronic case report for public health surveillance without extensive modifications.

## Methods

### Overview

This project was determined to be a quality improvement project by an automated review tool developed by the Medical University of South Carolina (MUSC) based on the federal definition of research; as such, it was exempted by MUSC from an institutional review board review. We developed and tested the PACER architecture using simulated data first. We then implemented this architecture at MUSC. During this pilot study, we did not enroll any health department, with the intention of testing the PACER architecture in a health care setting first. For these reasons, we simulated the role of the health department. The results were evaluated by MUSC, ensuring that patient data remained with the health system, and only the aggregate findings of the study were reported.

### Case Report Trigger Logic

Case reports need a trigger logic, which is a set of criteria that must be satisfied to qualify as a notifiable condition and create an automated case report. The PACER architecture uses incoming ELR to the health department as trigger criteria to generate queries into the EHR. The CSTE has developed standardized reporting definitions for notifiable conditions [19]. The goal of these position statements is to support a standard for case reporting from EHRs or other clinical care information systems. These position statements—which represent documentation and analysis regarding the case definition of the condition—are based on laboratory results, clinical observations (such as diagnosis), or a combination of both. The ELR infrastructure uses the CSTE’s position statements to qualify a case and create a report. ELR has seen widespread implementation since it was incentivized by the Centers for Medicare and Medicaid Services Meaningful Use program [20,21]. Electronic laboratory reports are a reliable and widely implemented method for case reporting. However, the major limitation is that electronic laboratory reports do not contain additional and important information needed to understand disease epidemiology.

### Design and Testing

**Figure 1:**
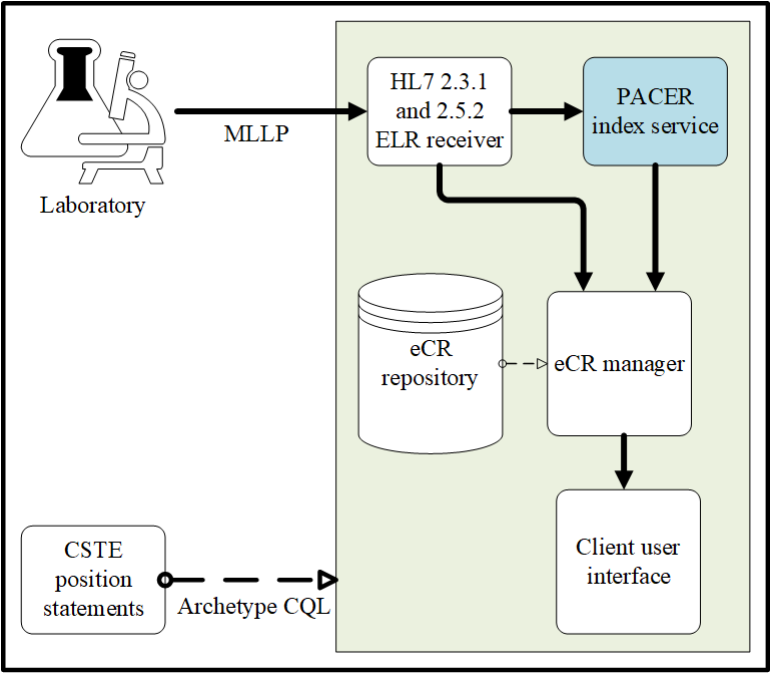
Electronic laboratory reports sent from laboratories are ingested by health departments and serve as the trigger for PACER electronic case reports. CSTE: Council for State and Territorial Epidemiologists; CQL: Clinical Quality Language; eCR: electronic case report; ELR: electronic laboratory reporting; HL7: health level 7; MLLP: Minimum Lower Level Protocol; PACER: Public Health Automated Case Event Reporting.

**Figure 2:**
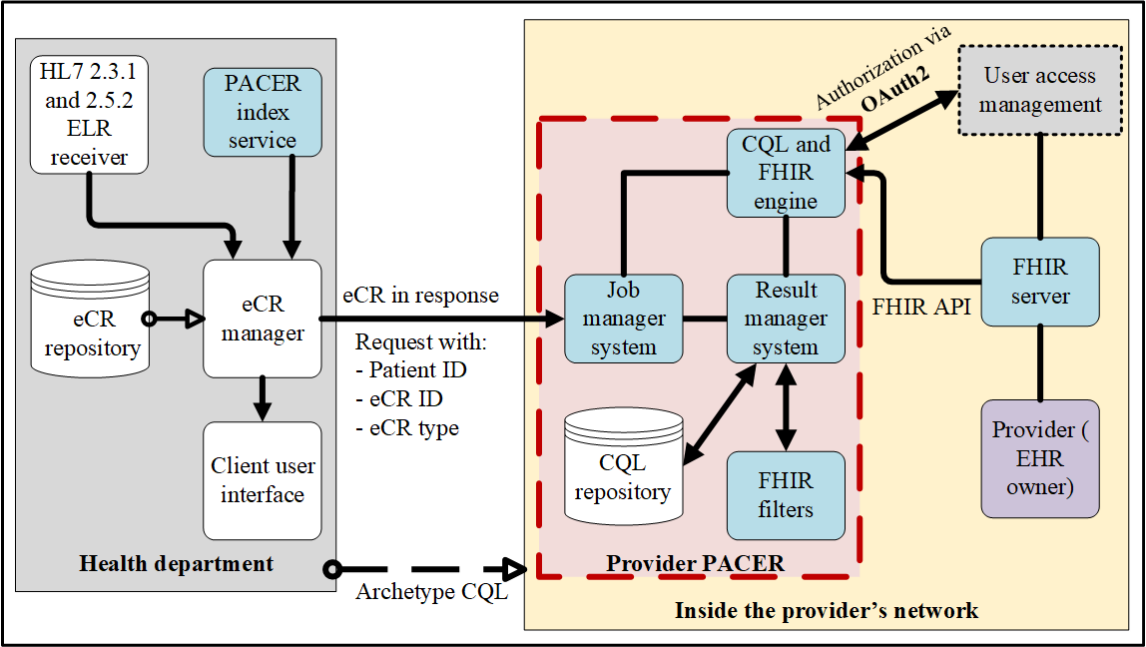
Detailed architecture of PACER. API: application programming interface; CQL: Clinical Quality Language; eCR: electronic case report; EHR: electronic health record; ELR: electronic laboratory reporting; FHIR: Fast Health Interoperability Resources; HL7: health level 7; OAuth2: OAuth 2.0 Authorization Framework; PACER: Public Health Automated Case Event Reporting.

**Figure 3:**
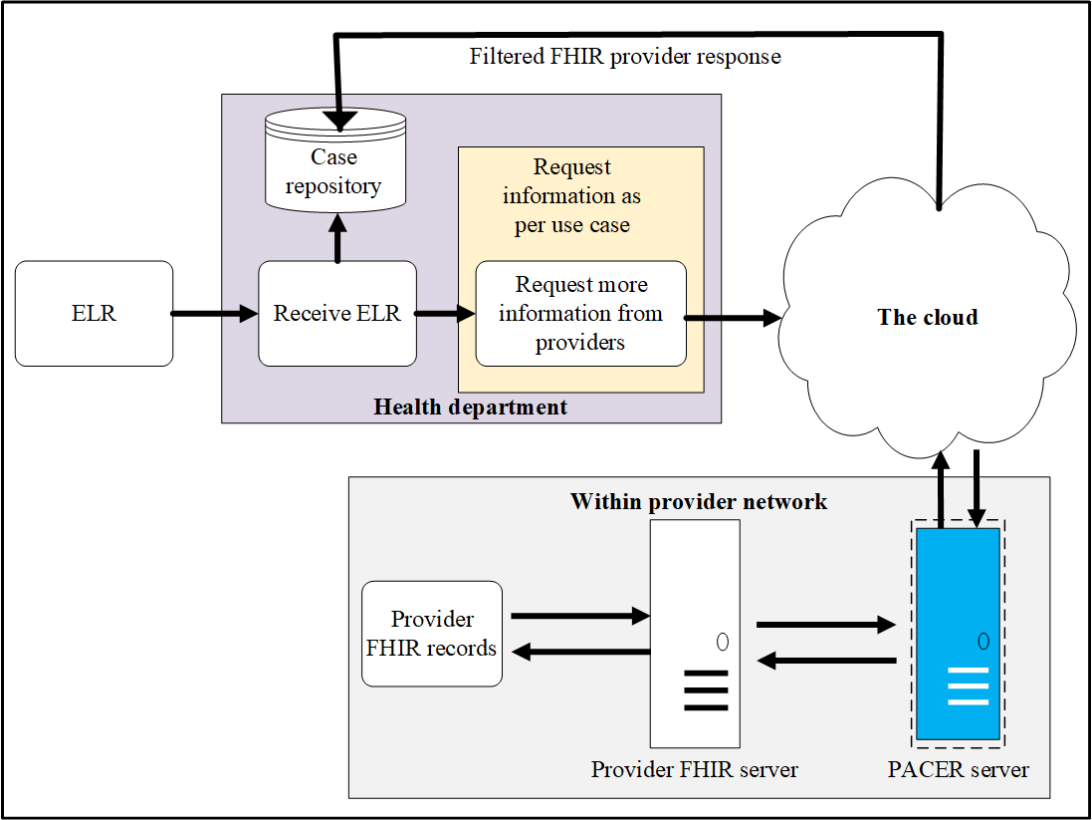
High-level architecture of PACER. ELR: electronic laboratory reporting; FHIR: Fast Health Interoperability Resources; PACER: Public Health Automated Case Event Reporting.

Design and testing were performed at the Georgia Tech Research Institute. During this phase, we used ELR messages entering a simulated health department as the initial trigger for PACER (Figure 1). Using the identifier for the patient and the clinical provider in the ELR message, a request for additional case information was sent to the clinical provider (Figure 2). The CQL logical statements were predetermined and provided before the request in the form of a CQL script. This script can be readily updated for the new report logic. A PACER server within the clinical provider’s network received and managed the query, as depicted in Figure 3. A CQL repository contained predetermined logical expressions as a CQL query syntax based on the typical request of the health department. On the basis of this CQL, a FHIR application programming interface (API) queried a FHIR server connected to the provider’s server, which is, in turn, connected to the EHR. This CQL engine can also integrate the standard and local terminologies. As not all FHIR systems adhere to the same standard codes, PACER was designed to have local codes and queries against the FHIR server. However, in the previous pilot study [18], it was found that this mechanism extracted a lot of redundant data not relevant to that particular reportable condition. In the current architecture, PACER includes a FHIR-based translation API service between the standard and local codes. Clinical providers can construct the local to standard mapping information in a comma-separated values format and load it to the translation API service, which can then be used to translate standard code to local code and vice versa during a CQL over FHIR operation. We also designed a *FHIR Filter*—an API service—which filters FHIR resources. This ensured that only those data elements predetermined by the provider were retrieved for the case report, and all irrelevant data, such as clinical observations and treatment information from unrelated clinical visits, were removed. This mechanism (Figure 2) ensured that the provider systems have complete control over the data extracted from the EHR and are consistent with the case reporting requirements. Filtered information, such as demographics, clinical observations, and treatment information, were retrieved from the EHR and then sent back to the simulated health department as an electronic case report, where it was received and stored in the eCR repository.

This process could potentially be replicated for any other notifiable conditions listed in the CSTE position statements or other similar standards. The PACER server is placed within the provider’s network firewall and connects to the FHIR servers of the EHR. This architecture ensures that control over the data extraction and filtering mechanism is entrusted to the providers themselves. Public health departments may only request and receive reports using preapproved queries, thereby avoiding the risk of data overreach or gathering information that is extraneous to the notifiable condition. PACER can be deployed on any internet-enabled server, either on premise or on the cloud. PACER can connect to multiple EHRs simultaneously and can extract case-related information from them.

### Pilot Deployment

We deployed PACER at the MUSC Health System by using the architecture shown in Figure 3. We continued to use the Georgia Tech Research Institute–simulated public health department (GTHD) server for the pilot study to ensure that all components were working. The MUSC configured their gateway proxy server to accept API calls from the GTHD server. The CSTE position statements for chlamydia [22] were translated into a CQL query syntax. This incoming positive chlamydia electronic laboratory report triggered an eCR request from the simulated public health department server.

**Figure 4:**
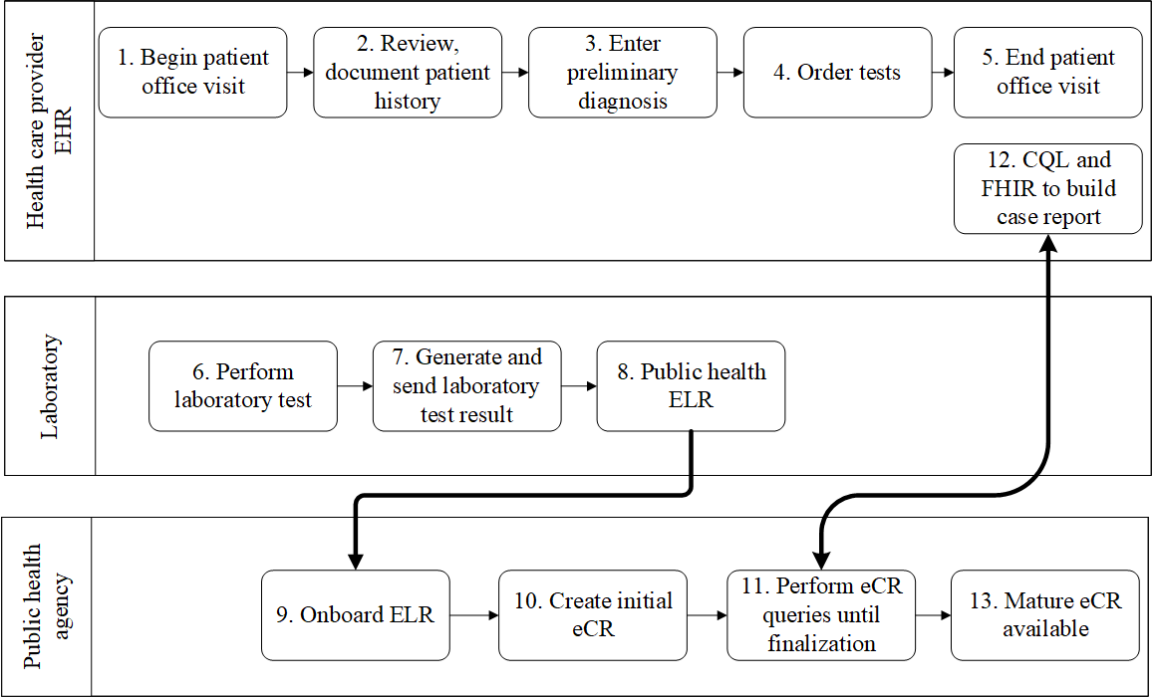
Laboratory, public health agency, and health care provider workflow to generate case reports using Public Health Automated Case Event Reporting. CQL: Clinical Quality Language; eCR: electronic case report; EHR: electronic health record; ELR: electronic laboratory reporting; FHIR: Fast Health Interoperability Resources.

First, full end-to-end testing was performed using synthetic data in the form of ELR sent to the GTHD server, which triggered the eCR manager (Figure 1) to send a request to the MUSC PACER server endpoint configured to use the synthetic FHIR data (Figure 2). From the ELR message, GTHD was able to send the PACER CQL request and successfully receive information to be incorporated into the initial case report, including information unavailable in the original ELR HL7 v2 message. This successfully demonstrated the workflow in Figure 4 from steps 7 to 13. After connectivity testing was completed, PACER was reconfigured to connect to the MUSC data assets. MUSC maintains a research FHIR server (SmileCDR) that contains a subset of its Epic EHR and supports external queries for FHIR resources. This server was used to respond to PACER queries. The outgoing connection was turned off to maintain privacy and confidentiality. Instead, the request was sent to PACER internally. The resulting electronic case reports were stored in a local MUSC environment and were analyzed locally.

### Evaluation Metrics

We evaluated the data elements extracted by PACER in the form of eCR, triggered by confirmed positive chlamydia laboratory results over an 8-week study period. The CQL query used in the pilot study was designed to obtain relevant data on patient diagnoses, symptoms, medications, and demographics of patients with chlamydia. We chose chlamydia because it was the most commonly reported condition, with more than 1.7 million cases reported in 2018 [23]. All evaluations and data analyses were performed at MUSC, the health system where PACER was deployed, and only the aggregate findings were reported by MUSC staff. The number of actual chlamydia cases and the number of electronic case reports created were compared to determine the success of the pilot. The completeness of case reports was also evaluated by assessing the presence of diagnoses, symptoms, medications, and demographics in each electronic case report. These same data were evaluated to test whether data relevant only to that particular event of chlamydia diagnosis were filtered by PACER. We also evaluated the type of data elements that PACER eCR extracts compared with ELR. The project was evaluated based on the accessible data from the MUSC FHIR server.

## Results

By using 8 weeks of laboratory data, 117 positive chlamydia laboratory tests triggered an electronic case report request through the PACER system deployed at MUSC. PACER successfully created 100% (117/117) of the electronic case reports containing data elements specified in the CQL. As shown in Table 1, PACER was able to obtain all key data elements for the majority of patients. In addition to routine demographic data, PACER was able to obtain the clinical diagnosis of 64.9% (76/117) of patients, treatment medications for chlamydia in 72.6% (85/117) of patients, and information on sexually transmitted infection symptoms in 70.9% (83/117) of patients.

**Table 1:** Patient data retrieved from electronic case reports in electronic health records (N=117).

Data elements	Case reports that contained the data elements, n (%)
Demographics	116 (99.1)
Medications	85 (72.6)
Symptoms	83 (70.9)
Diagnoses	76 (65)

Table 2 provides a perspective on the types of data available in ELR and the PACER eCR. As noted, electronic laboratory reports report laboratory results but with limited demographic data and no information on conditions, medications, or symptoms. Electronic laboratory reports report confirmed positive laboratory findings, which easily met the reporting criteria, and can be successfully used as a trigger for eCR. Complementing the electronic laboratory report with PACER enhanced case reports by extracting critical additional data elements, including medications, symptoms, and clinical diagnoses.

**Table 2:** Comparison between the case reports from ELR^a^ and PACER^b^.

Data elements	Case report from ELR	Case report from PACER
Patient demographics	Limited—as available in the laboratory order	Nearly complete if allowed by providers
Laboratory results	Available	Available
Conditions	Not available	Available
Medications	Not available	Available
Extensibility	Limited by ELR specification	Can be extended by defining additional resources in CQL^c^

^a^ELR: electronic laboratory reporting.

^b^PACER: Public Health Automated Case Event Reporting.

^c^CQL: Clinical Quality Language.

## Discussion

### Principal Findings

We piloted the use of an electronic case report architecture combining the widespread adoption and reliability of ELR with the robustness of EHR data through a system leveraging the rapidly emerging FHIR standard. The CQL and permissions for data access can change dynamically during outbreaks. Thus, an important advantage of the PACER design is its ability to respond to the evolving needs of clinical data for emerging infectious diseases such as COVID-19. This architecture was able to provide additional sexually transmitted infection–relevant clinical data on diagnoses, symptoms, and medications in 65% (76/117), 70.9% (83/117), and 72.6% (85/117) of patients, respectively. Moreover, once the initial queries were approved, these data were captured without human intervention, minimizing the burden of manual chart review. The FHIR query can only bring data available in the EHRs at the time the query reaches the EHR. There could be other factors responsible for the unavailability of the EHR data, such as laboratory tests stored in local codes and not LOINC. Some of this could be mitigated by sending FHIR queries on certain periodic intervals, such as treatment information, which may only be available after a certain time lag post diagnosis. PACER clearly demonstrates advantages in enhancing electronic laboratory reports and C-CDA–based electronic case reports. PACER can extract important demographic, diagnoses, treatment, and other relevant case information that the electronic laboratory reports, by virtue of being reported out of laboratories, will never contain.

C-CDA–based eCR is triggered at a point in the continuum of clinical care [24], such as the initial visit where the case definition is met. Some local health jurisdictions mandate clinical providers to report cases within a definite period of the case being known or diagnosed. Typically, treatment information and other relevant data generated chronologically along the continuum of care will not be available at the time the electronic case report is generated. The PACER architecture remedies these issues. The ELR trigger satisfies the time-bound reporting mandate imposed by some jurisdictions. As ELR triggers FHIR queries at points that are chronologically further along the continuum of care, the PACER architecture can extract such data that may not be available at the time a case report is triggered. In addition, although the C-CDA format extracts a case report in its entirety every time it is triggered [24], the FHIR format allows the query for specific data points. Subsequent queries can be triggered at any time, seeking specific information, such as treatment or follow-up data from EHRs. Within the PACER architecture, the eCR manager handles this request, and multiple information requests can be queried at any point along the care continuum.

In the pilot of an earlier iteration of PACER, we discovered that the architecture extracted every record of the individual, including information that was not relevant to the reportable disease [18]. In this iteration, we added filters to the provider server to ensure that only relevant (and predetermined) information related to the reportable disease is extracted. The filtering of data within a FHIR resource is needed when the FHIR resource contains more data elements than required for public health case reporting. In conjunction with the CQL logic (predetermined between the clinical provider and health departments), this design prevented the release of any unrelated information but allowed for the secure and free flow of information for public health. PACER is an open-source architecture that is maintained on GitHub and is freely accessible by the public. We plan to collaborate with clinical providers, EHR service providers, and local and state health departments to make PACER more robust and easily accessible. We also continue to expand the use of PACER architecture for other public health uses.

### Limitations

There are several challenges in the current environment of PACER. First, the design of the clinical queries using CQL relies on understanding the local clinical codes used within the health systems, if applicable. To maintain the quality of eCR outputs over different provider systems, it is necessary to standardize to common code systems at the CQL level while enabling mapping at the individual provider level. This standardization can be facilitated by maintaining value sets that include medical codes used in provider systems and maps from local medical codes to standard codes. For the pilot study, we found that some local medical code mappings were missing and required additional manual effort to finalize the value sets. Complete and accurate mapping is a perennial challenge, and in some contexts, it is worth sacrificing *edge case* codes (ie, rarely used nonstandard codes) to create systems that are portable and generalizable. We also expect the recent formalization of US Core Data for Interoperability to have a positive impact on the ability to retrieve standardized concepts going forward.

The other major consideration of this study is the adoption of FHIR itself. FHIR are rapidly emerging, with more than 80% of US hospitals using a FHIR-capable EHR. Even then, the FHIR capability is not the same as the FHIR capacity. One of the limitations of this work was the use of a dedicated FHIR server that contains only a subset of the data available in the MUSC EHR. However, health systems are still working through the governance processes for how FHIR will be used in their environments, and thus, the coordination between health systems’ information technology departments and public health will be essential for successful automated reporting, as demonstrated here. For mitigating these challenges, PACER has been designed to be (1) highly protective of health system data with privacy controls and (2) flexible enough to support other health system operations and data query needs that may fall outside of public health reporting. This represents a possible *carrot* to health systems considering the implementation of a system but are cautious regarding dedicating information technology resources to a single use-case effort.

### Conclusions

Enhancing electronic laboratory reports with FHIR-based EHR data using an automated system such as PACER can expand access to relevant clinical data for public health reporting and decision-making. This method can extract important data to understand public health epidemiology and, in turn, inform policy and strategies to prevent and control sexually transmitted diseases in general and potentially aid in response to emerging infectious disease threats. Using ELR—or even eCR—as a trigger for FHIR-based queries that create additional electronic case reports with other relevant information is an adaptable and modular approach to enhancing case reporting. Clinical providers will benefit from this platform by automating the reporting of notifiable diseases and eliminating the burden of manual reporting. More importantly, clinical providers will have complete control of what data are reported to public health agencies. This is achieved through FHIR-based filters and can be further modified by the clinical providers at any time. PACER is EHR agnostic and is installed and invoked in a centralized manner, eliminating the need for multiple installations at every instance of an EHR. This approach not only alleviates the burden of manual reporting from providers but also public health departments get timely, accurate (from the EHR—the source of the clinical interaction), and more complete and detailed information on a case. As the type of information that can be extracted is predetermined (using CQL-based logic), any type of information can be retrieved as long as both the clinical provider and public health agencies agree on them. This will lead to efficient case investigations with essential information that is automatically populated. Traditionally, public health has struggled with getting information, such as treatment, medications, and symptoms. This approach provided additional information that will help public health better understand disease epidemiology. This architecture can be implemented for any other notifiable condition that has CSTE position statements translated into a logical format or even used for distributed data collection for an emerging infection such as COVID-19. This design emphasizes giving complete control of patient data to clinical providers, with the ability to filter what information is sent to public health agencies as part of case reports for notifiable diseases. This architecture is a step toward bridging public health with health care systems and can be used to develop a *query-based* system to gain important and relevant information related to public health priority. Future work will include expanding the pilot study to multiple facilities in local public health jurisdictions and engaging with the health systems within those jurisdictions to set up PACER. In addition, we are currently expanding PACER to build a FHIR-based syphilis registry.

## Abbreviations

API: application programming interface
C-CDA: Consolidated Clinical Document Architecture
CQL: Clinical Quality Language
CSTE: Council for State and Territorial Epidemiologists
eCR: electronic case reporting
EHR: electronic health record
ELR: electronic laboratory reporting
FHIR: Fast Health Interoperability Resources
GTHD: Georgia Tech Research Institute–simulated public health department
HL7: health level 7
LOINC: Logical Observation Identifiers Names and Codes
MUSC: Medical University of South Carolina
PACER: Public Health Automated Case Event Reporting
